# Therapeutic Potential and Challenges of Targeting Receptor Tyrosine Kinase ROR1 with Monoclonal Antibodies in B-Cell Malignancies

**DOI:** 10.1371/journal.pone.0021018

**Published:** 2011-06-15

**Authors:** Jiahui Yang, Sivasubramanian Baskar, Ka Yin Kwong, Michael G. Kennedy, Adrian Wiestner, Christoph Rader

**Affiliations:** 1 Experimental Transplantation and Immunology Branch, Center for Cancer Research, National Cancer Institute, National Institutes of Health, Bethesda, Maryland, United States of America; 2 Hematology Branch, National Heart, Lung, and Blood Institute, National Institutes of Health, Bethesda, Maryland, United States of America; The University of Birmingham, United Kingdom

## Abstract

**Background:**

Based on its selective cell surface expression in chronic lymphocytic leukemia (CLL) and mantle cell lymphoma (MCL), receptor tyrosine kinase ROR1 has recently emerged as a promising target for therapeutic monoclonal antibodies (mAbs). To further assess the suitability of ROR1 for targeted therapy of CLL and MCL, a panel of mAbs was generated and its therapeutic utility was investigated.

**Methodology and Principal Findings:**

A chimeric rabbit/human Fab library was generated from immunized rabbits and selected by phage display. Chimeric rabbit/human Fab and IgG1 were investigated for their capability to bind to human and mouse ROR1, to mediate antibody-dependent cellular cytotoxicity (ADCC), complement-dependent cytotoxicity (CDC), and internalization, and to agonize or antagonize apoptosis using primary CLL cells from untreated patients as well as MCL cell lines. A panel of mAbs demonstrated high affinity and specificity for a diverse set of epitopes that involve all three extracellular domains of ROR1, are accessible on the cell surface, and mediate internalization. The mAb with the highest affinity and slowest rate of internalization was found to be the only mAb that mediated significant, albeit weak, ADCC. None of the mAbs mediated CDC. Alone, they did not enhance or inhibit apoptosis.

**Conclusions and Significance:**

Owing to its relatively low cell surface density, ROR1 may be a preferred target for armed rather than naked mAbs. Provided is a panel of fully sequenced and thoroughly characterized anti-ROR1 mAbs suitable for conversion to antibody-drug conjugates, immunotoxins, chimeric antigen receptors, and other armed mAb entities for preclinical and clinical studies.

## Introduction

Chronic lymphocytic leukemia (CLL) is characterized by the presence of a monoclonal B-cell population with a count of >5,000 cells/µL in the peripheral blood [1], [2]. In the United States of America, CLL is the most common leukemia with roughly 15,000 new cases and 5,000 deaths per year. Whereas approximately half of CLL patients have an indolent clinical course that does not require treatment for many years, a more aggressive clinical course that necessitates treatment within a few years is diagnosed for the other half. These differences in clinical course correlate with molecular markers, including the mutational status of the surface immunoglobulins and the expression of intracellular tyrosine kinase ZAP-70. Despite a typically lower expression of CD20 compared to normal B cells, the combination of fludarabine and cyclophosphamide (FC) with the chimeric mouse/human anti-CD20 IgG1 monoclonal antibody (mAb) rituximab (FCR) [3] has become standard first-line treatment that was approved by the Food and Drug Administration (FDA) in 2010. In addition, alemtuzumab, a humanized anti-CD52 IgG1 mAb, was FDA-approved in 2001 as single agent for CLL therapy. Alemtuzumab is frequently used for second-line treatment, but is ineffective in CLL patients with bulky lymphadenopathy. In 2009, fully human IgG1 mAb ofatumumab, which binds to a CD20 epitope different from rituximab, was FDA-approved for treating CLL patients refractory to fludarabine and alemtuzumab.

With three mAbs approved for CLL out of a current total of eleven mAbs approved for cancer therapy [4], CLL clearly has become a preferred indication for mAbs [5]. Biologically, this can be explained by the accessibility of leukemia cells in circulation and the availability of effector cells and proteins that mediate antibody-directed cellular cytotoxicity (ADCC) and complement-dependent cytotoxicity (CDC), respectively. In addition, the only potentially curative treatment available for CLL patients is allogeneic hematopoietic stem cell transplantation which may involve an endogenous antibody response against CLL cell surface antigens [6]. Although targeting CD20 and CD52 with mAbs can increase progression-free survival and overall survival of CLL patients, immune suppression caused by the expression of these cell surface antigens on normal B cells and other leukocytes can trigger serious infectious complications such as hepatitis B virus reactivation and progressive multifocal leukoencephalopathy following rituximab [7] and cytomegalovirus reactivation and pneumocystis pneumonia following alemtuzumab [8] treatments. These adverse events provide a rationale for the discovery of cell surface antigens with restricted expression on CLL cells and for the development of mAbs that selectively target them. It is anticipated that such mAbs would facilitate a precise intervention without concomitant immune suppression.

Mantle cell lymphoma (MCL) [9] is an incurable subtype of non-Hodgkin lymphoma with roughly 4,000 new cases and 2,000 deaths per year in the United States of America and a median survival of only 3–5 years. Approximately 25% of MCL patients have a leukemic component. Interestingly, MCL and CLL cells share the general immunophenotype CD5+ CD19+ CD20+ IgD+ IgM+ which is not found in other B-cell malignancies; however, MCL cells are generally CD23− whereas CLL cells are CD23+. In contrast to CLL, a common genetic abnormality characterizes MCL. Chromosomal translocation t(11;14) places the cyclin D1 gene under the control of the immunoglobulin heavy chain promoter, promoting entry into the cell cycle. MAb therapy is not well established for MCL [10]. Whereas the addition of rituximab to chemotherapy, such as CHOP (cyclophosphamide/doxorubicin/vincristine/prednisone), has become standard first-line treatment for non-Hodgkin lymphoma with significant benefit compared to chemotherapy alone in follicular lymphoma and diffuse large B cell lymphoma, only very modest benefits were found for MCL [11]. Although radioimmunotherapy with the FDA-approved mAbs ^90^Y-labeled ibritumomab tiuxetan and ^131^I-labeled tositumomab, which like rituximab recognize CD20, appears more promising [12], there clearly is a need for innovative drug and target discovery for mAb therapy of MCL.

A candidate cell surface antigen for selective targeting of both CLL and MCL is the receptor tyrosine kinase ROR1. The upregulation of ROR1 mRNA in CLL was discovered by gene expression profiling [13], [14]. ROR1 and the only other member of the ROR family, ROR2, are type I membrane proteins that share 58% amino acid sequence identity and a unique extracellular region with one immunoglobulin (Ig), one frizzled (Fz), and one kringle (Kr) domain followed by a transmembrane region and an intracellular region that includes a tyrosine kinase domain [15], [16]. Studies in rodents revealed that ROR1 and ROR2 are broadly expressed in developing organs during embryogenesis and tightly downregulated after birth [15], [17], [18]. Functionally, there is growing evidence that ROR1 and ROR2 mediate noncanonical Wnt signaling by serving as receptors for the ligand WNT5A [19]. Due to their recently discovered upregulation in certain hematologic and solid malignancies [19], ROR1 and ROR2 may be considered oncofetal proteins. We and others have shown the restricted, uniform, and constitutive cell surface expression of ROR1 in CLL [20], [21], [22]. Cell surface expression of ROR1 was also demonstrated in both primary MCL cells and MCL cell lines [23]. At a density of 10^3^ to 10^4^ molecules per cell, ROR1 revealed selective expression on the CLL cell surface independent of the noted molecular correlates that predict the clinical course. Normal B cells, other normal blood cells, and normal adult tissues did not express ROR1 at detectable levels. A portion of ROR1 was internalized when bound by polyclonal antibodies (pAbs), and only low concentrations of soluble ROR1 were detected in sera from CLL patients [20]. Collectively, these findings suggested that ROR1 would be an excellent target for mAbs and other antibody derivatives. Given the relatively large extracellular region of ROR1 and its organization into three independent domains, it will be critical to identify epitopes that are suitable for mediating ADCC, CDC, internalization, and potential functional interference.

We previously established the generation of chimeric rabbit/human Fab libraries and their selection by phage display as a robust method for the generation of panels of mAbs that bind with high specificity and affinity to different epitopes of a variety of antigens [24], [25], [26], [27], [28]. Here we use this method to select a panel of Fab with rabbit variable domains and human constant domains to different epitopes in all three extracellular domains of human ROR1. As chimeric rabbit/human Fab can be converted to chimeric rabbit/human IgG1 with fully human Fc domains, this strategy readily provides reagents suitable for ADCC and CDC studies with primary human target and effector cells. Moreover, although various humanization strategies are readily available for rabbit variable domains [24], [29], [30], chimeric rabbit/human IgG1 may already be suitable for clinical studies without further antibody engineering.

## Results

### Selection of chimeric rabbit/human Fab by phage display

**Figure 1:**
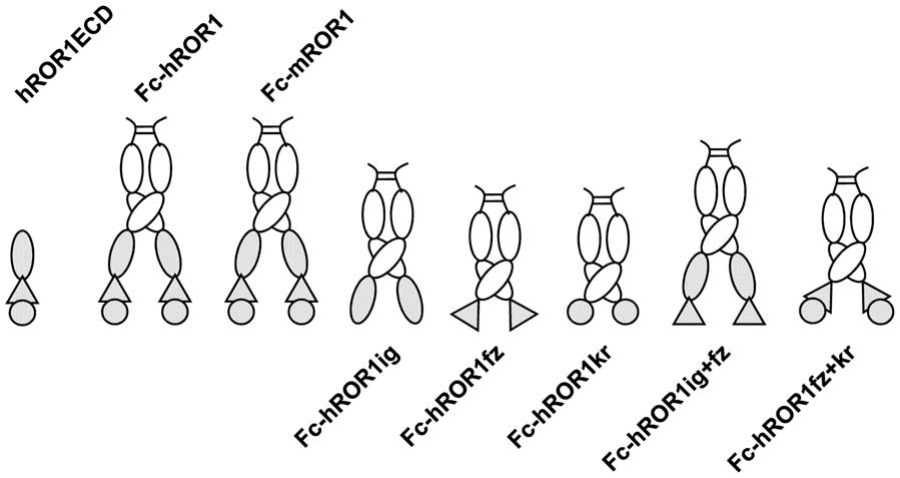
Recombinant ROR1 proteins. Eight recombinant proteins with different compositions of the three extracellular domains of ROR1 were constructed for this study and expressed in HEK 293F cells. ROR1 Ig, Fz, and Kr domains are shown as oval, triangle, and circle, respectively (gray). Seven recombinant proteins, including two that included all three extracellular domains of human (h) and mouse (m) ROR1 as well as five with just one or two extracellular domains of human ROR1, were generated as C-terminal fusion to the dimeric Fc domain of human IgG1 (white) and purified by Protein A affinity chromatography. Another recombinant protein composed of the three extracellular domains of human ROR1 alone (hROR1ECD) was purified by ion exchange and gel filtration chromatography.

**Figure 2:**
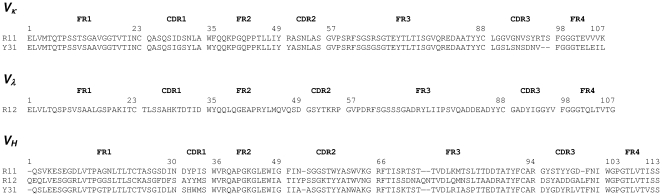
Amino acid sequences of R11, R12, and Y31. The amino acid sequence alignment of the rabbit variable domains (V_κ_, V_λ_, and V_H_) of chimeric rabbit/human Fab clones R11, R12, and Y31 is shown with framework regions (FR) and complementarity determining regions (CDR) using Kabat numbering [46].

The three extracellular domains of human ROR1 were expressed alone (hROR1ECD) or as fusion protein with the Fc domain of human IgG1 (Fc-hROR1) (**Fig. 1**). Purified Fc-hROR1 and hROR1ECD were used to immunize and boost two groups of rabbits of the b9 allotype which we previously had shown to be superior for the generation and selection of chimeric rabbit/human Fab libraries [25]. RNA from spleens and bone marrow of the two groups of rabbits, which differed with respect to immunogen and adjuvant, were used to generate two independent chimeric rabbit/human Fab libraries and select them by phage display on immobilized hROR1ECD and Fc-hROR1 as described [28]. As summarized in **Table 1**, seven different chimeric rabbit/human Fab clones that bound to hROR1ECD were identified. Among these, three clones also bound to Fc-hROR1 and cell surface human ROR1 expressed by stably transfected human embryonic kidney (HEK) 293F cells [31]. These three clones (designated R11, R12, and Y31) were therefore pursued further. The diverse amino acid sequences of both frameworks and complementarity determining regions of the rabbit variable domains of R11, R12, and Y31 (**Fig. 2**) revealed the usage of unrelated V_κ_ (R11, Y31), V_λ_ (R12), and V_H_ germlines, suggesting the recognition of different epitopes on human ROR1.

**Table 1:** Panel of chimeric rabbit/human Fab selected by phage display.

Clone^1^	Library	Panning rounds		Repeated clones	Binding		
		hROR1ECD	Fc-hROR1		hROR1ECD^2^	Fc-hROR1^2^	HEK 293F/hROR1^3^
**R11**	R	4	0	26/31	++	++	+
**R12**	R	4	0	1/31	++	++	++
**Y4**	Y	4	0	2/31	++	−	−
**Y13**	Y	4	0	14/31	++	−	−
**Y14**	Y	4	0	2/31	+	−	−
**Y27**	Y	4	0	13/31	++	−	−
**Y31**	Y	3	1	4/4	+	+	+

1 Defined by unique DNA fingerprint and sequence.

2 As measured by ELISA.

3 As measured by flow cytometry.

### Specificity and epitope mapping of chimeric rabbit/human Fab and IgG1

**Figure 3:**
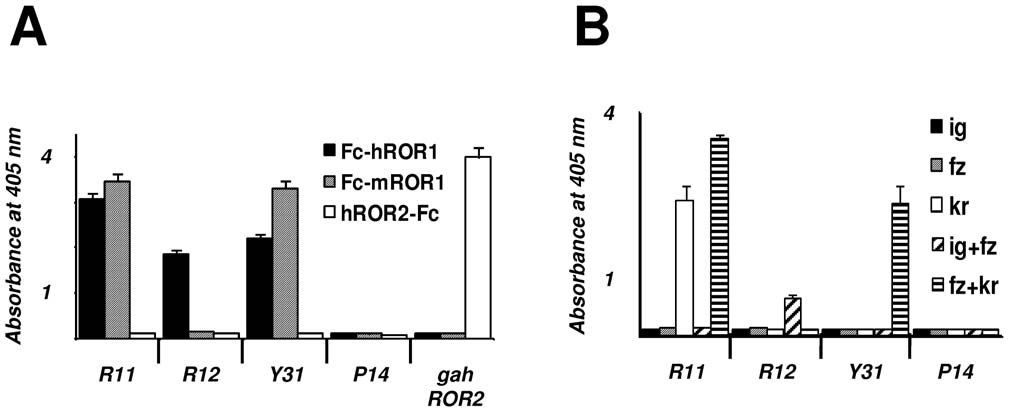
Specificity and epitope mapping studies by ELISA. (**A**) The binding of chimeric rabbit/human IgG1 R11, R12, Y31, and P14 to immobilized human ROR1 (Fc-hROR1), mouse ROR1 (Fc-mROR1), and human ROR2 (hROR2-Fc) was analyzed by ELISA using goat anti-human kappa pAbs conjugated to HRP. The binding of goat anti-human ROR2 pAbs (gahROR2) was detected with donkey anti-goat IgG (H+L) pAbs conjugated to HRP. (**B**) Using the same procedure, the epitopes of IgG1 R11, R12, and Y31 were mapped with five immobilized Fc fusion proteins that consisted of just one or two extracellular domains of human ROR1 (see **Fig. 1**). These were Fc-hROR1ig (ig), Fc-hROR1fz (fz), Fc-hROR1kr (kr), Fc-hROR1ig+fz (ig+fz), and Fc-hROR1fz+kr (fz+kr). Columns indicate mean values and error bars indicate standard deviation values of side-by-side triplicates.

**Figure 4:**
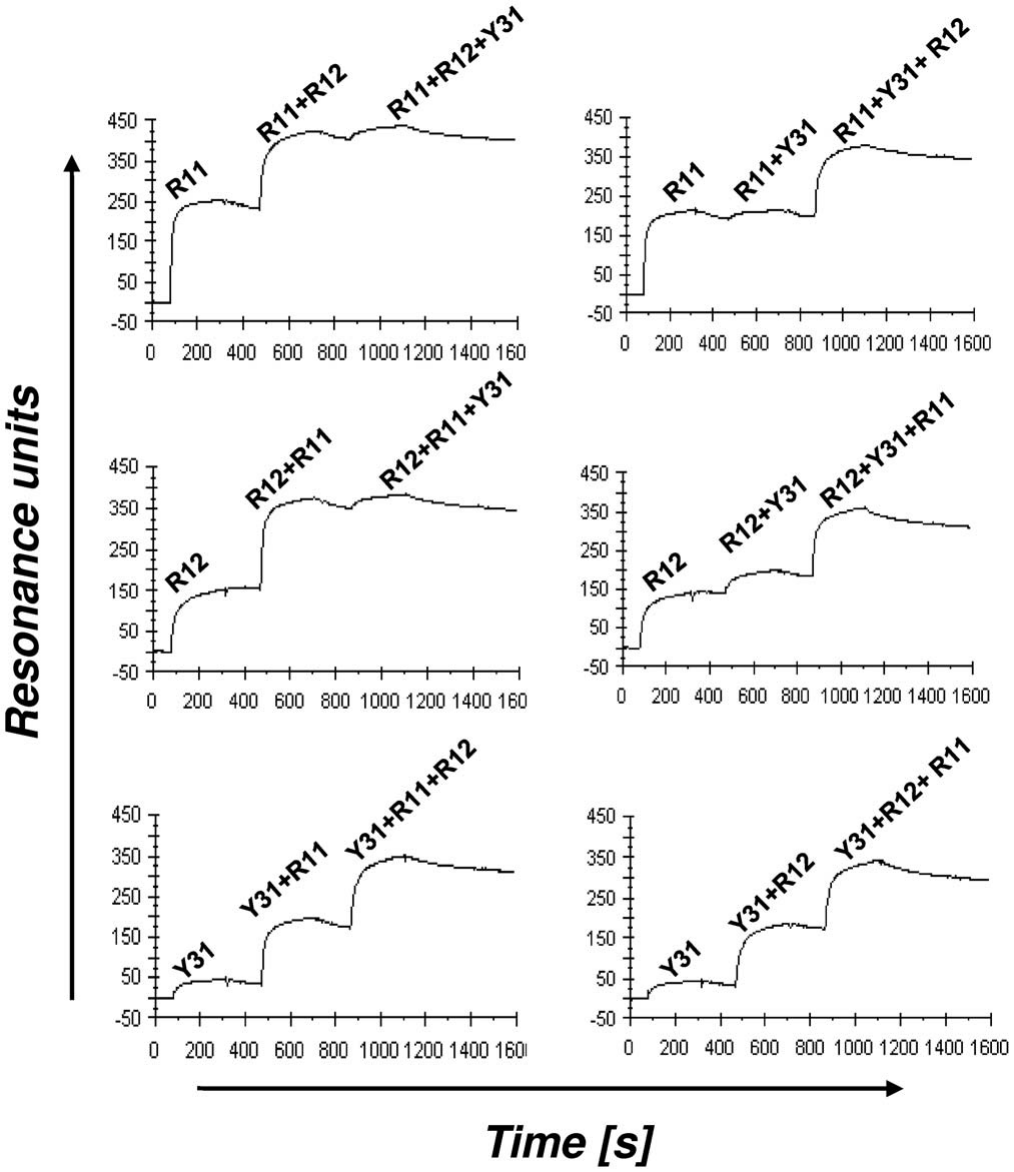
Epitope mapping studies by surface plasmon resonance. Shown are Biacore ×100 sensorgrams obtained for the binding of IgG1 R11, R12, and Y31 to immobilized Fc-hROR1. IgG1 were injected in different orders and mixtures to identify independent and overlapping epitopes. Resonance unit (*y* axis) increases that exceeded the values found for IgG1 R11, R12, and Y31 alone indicated independent epitopes that allow simultaneous binding. For example, the increase found for the combination of IgG1 R11 and R12 (top left and middle left graph) exceeded the values found for R11 and R12 alone, indicating that R11 and R12 can bind simultaneously to human ROR1. By contrast, the increase found for the combination of IgG1 Y31 and R11 (top right and bottom left graph) did not exceed the values found for R11 and Y31 alone, indicating overlapping epitopes. The simultaneous binding of IgG1 R12 and Y31 can be seen in the middle right graph. The *x* axis depicts the time in seconds (s).

Both chimeric rabbit/human Fab and chimeric rabbit/human IgG1 formats were used for further characterizing the three selected mAbs in terms of specificity and affinity. The specificity of purified Fab and IgG1 was probed by ELISA with an extended panel of recombinant ROR1 proteins that included Fc-hROR1, its mouse analogue Fc-mROR1, and five Fc fusion proteins with just one or two extracellular domains of human ROR1 (**Fig. 1**). Also included was commercially available hROR2-Fc. Chimeric rabbit/human Fab and IgG1 P14 against NgR2 [27] was used as negative control. Fab (data not shown) and IgG1 (**Fig. 3**) revealed identical binding patterns. As shown in **Fig. 3A**, IgG1 R11, R12, and Y31 bound to human ROR1, but not to human ROR2. In addition, IgG1 R11 and Y31 were found to be cross-reactive with mouse ROR1. The binding of IgG1 R11, R12, and Y31 to just one or two extracellular domains of human ROR1 (**Fig. 3B**) confirmed the recognition of three different epitopes. In selectively recognizing Fc-hROR1kr and Fc-hROR1fz+kr, IgG1 R11 was the only mAb that mapped to a single domain. By contrast, IgG1 R12 and Y31 selectively recognized Fc-hROR1ig+fz and Fc-ROR1fz+kr, respectively, but not any of the single domains, suggesting that the epitopes of these mAbs are located in the region that links two neighboring domains, i.e. at the conjunction of Ig and Fz domains in case of R12 and at the conjunction of Fz and Kr domains in case of Y31. Alternatively, they may bind to conformational epitopes that require the presence of these two neighboring domains. Importantly, the three epitopes of IgG1 R11, R12, and Y31 were found to cover a large portion of the extracellular region of human ROR1, allowing us to investigate the therapeutic implications of membrane distal and proximal binding of anti-ROR1 mAbs. The independence of the three epitopes was also analyzed by surface plasmon resonance using a Biacore ×100 instrument. For this, Fc-hROR1 was immobilized on the sensor chip and IgG1 R11, R12, and Y31 were injected in different order. As shown in **Fig. 4**, IgG1 R11 and R12 were found to bind simultaneously and independently to Fc-hROR1 regardless of the sequence of injection. By contrast, IgG1 R11, but not IgG1 R12, was found to block the binding of IgG1 Y31 when injected first or compete with the binding of IgG1 Y31 when injected second. Using Fab instead of IgG1 in this study gave the same qualitative data (data not shown). Collectively, these findings suggested that the epitopes of R11 in the Kr domain and Y31 at the conjunction of Fz and Kr domains partially overlap whereas R12 binds to an independent epitope at the conjunction of Ig and Fz domains. The simultaneous binding of IgG1 R11 and R12 to Fc-hROR1 encouraged an investigation of their therapeutic potential in combination. A mixture of two anti-EGFR mAbs directed against independent epitopes was recently shown to convey superior activity in preclinical studies when compared to either antibody alone [32].

### Affinity and avidity of chimeric rabbit/human Fab and IgG1

**Figure 5:**
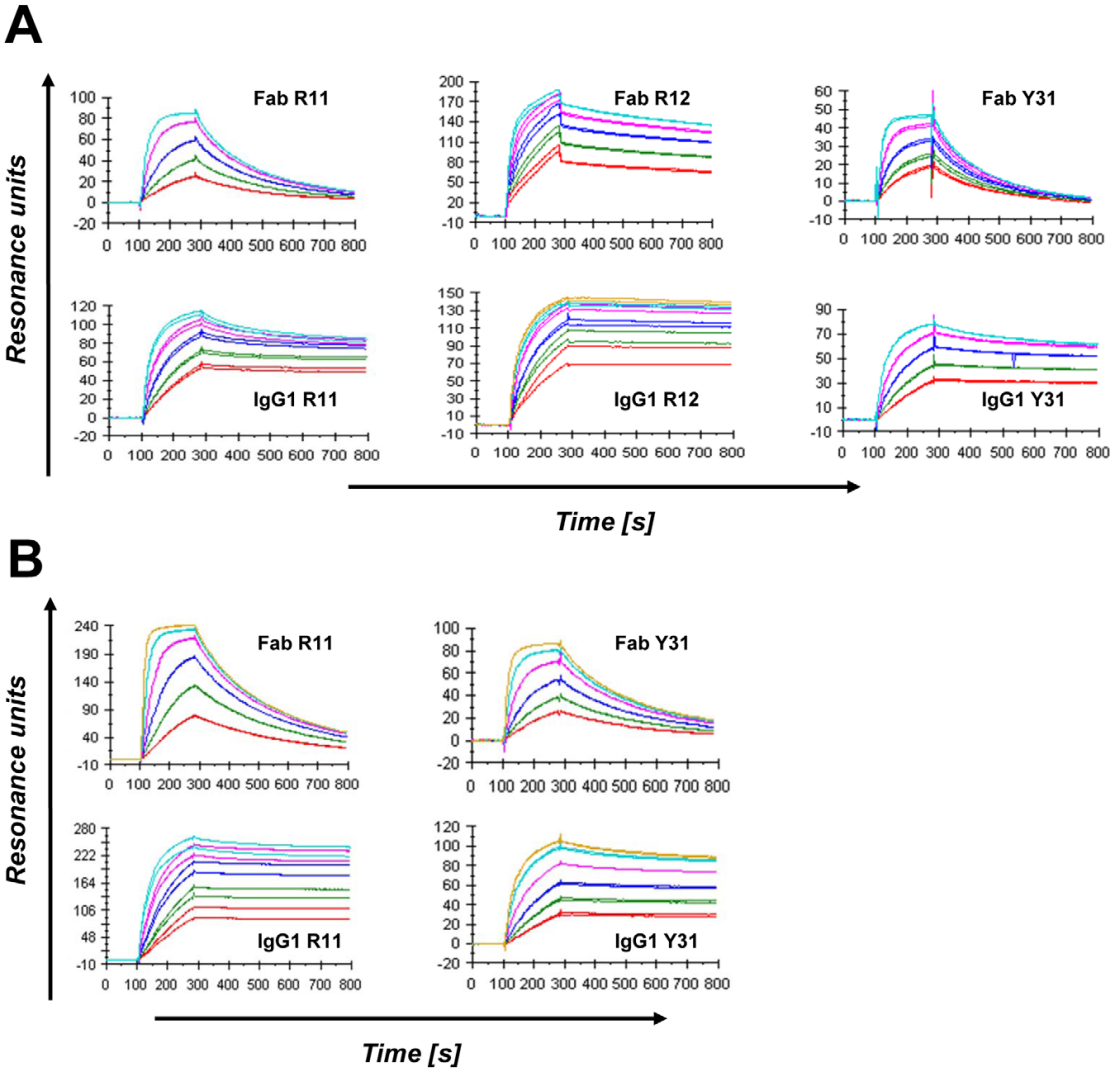
Affinity measurements by surface plasmon resonance. Shown are Biacore ×100 sensorgrams obtained for the binding of Fab and IgG1 R11, R12, and Y31 to immobilized Fc-hROR1 (**A**) and Fc-mROR1 (**B**) after instantaneous background depletion. The mAbs were injected at five or six different concentrations (shown in different colors) ranging from 1.5 to 100 nM. Each concentration was tested in duplicates.

Surface plasmon resonance was also used to measure the affinity and avidity of the three selected mAbs in Fab and IgG1 formats, respectively (**Table 2** and **Fig. 5**). Fab R12 was found to be the strongest binder with an affinity of 0.56 nM to Fc-hROR1. Fab R11 and Y31 revealed affinities of 2.7 and 8.8 nM, respectively. These differences were largely due to an approximately twenty fold slower dissociation rate of Fab R12 which, in case of Fab R11, was partially compensated by a faster association rate. Conversion from monovalent Fab to bivalent IgG1 increased the virtual affinity of R11, R12, and Y31 by factor 14, 5, and 12, respectively; all three IgG1 revealed subnanomolar avidity. Confirming the ELISA data, R11 and Y31 revealed comparable affinities and avidities for Fc-hROR1 and Fc-mROR1, suggesting that their epitopes are entirely conserved between human and mouse ROR1. By contrast, R12 did not reveal detectable binding to Fc-mROR1.

**Table 2:** Affinities and avidities of R11, R12, and Y31 in Fab and IgG1 formats.^1^

MAb	Antigen	*k*_on_(10^5^) (M^−1^s^−1^)	*k*_off_(10^−4^) (s^−1^)	*K*_d_(nM)
**Fab R11**	Fc-hROR1	20.4	54.7	2.7
	Fc-mROR1	16.9	50.4	3.0
**IgG1 R11**	Fc-hROR1	19.4	3.6	“0.19”
	Fc-mROR1	9.9	3.0	“0.30”
**Fab R12**	Fc-hROR1	5.5	3.1	0.56
	Fc-mROR1	no binding	no binding	no binding
**IgG1 R12**	Fc-hROR1	5.5	0.62	“0.11”
	Fc-mROR1	no binding	no binding	no binding
**Fab Y31**	Fc-hROR1	8.5	75.2	8.8
	Fc-mROR1	9.1	38.3	4.2
**IgG1 Y31**	Fc-hROR1	4.9	3.5	“0.71”
	Fc-mROR1	5.4	2.4	“0.44”

1 Fc-hROR1 or Fc-mROR1 were immobilized on the sensor chip. Association (*k*_on_) and dissociation (*k*_off_) rate constants were calculated using Biacore ×100 evaluation software. The equilibrium dissociation constant (*K*_d_), which is shown in quotes for IgG1 to indicate the contribution of the avidity effect, was calculated from *k*_off_/*k*_on_.

### Cell surface binding of chimeric rabbit/human IgG1

**Figure 6:**
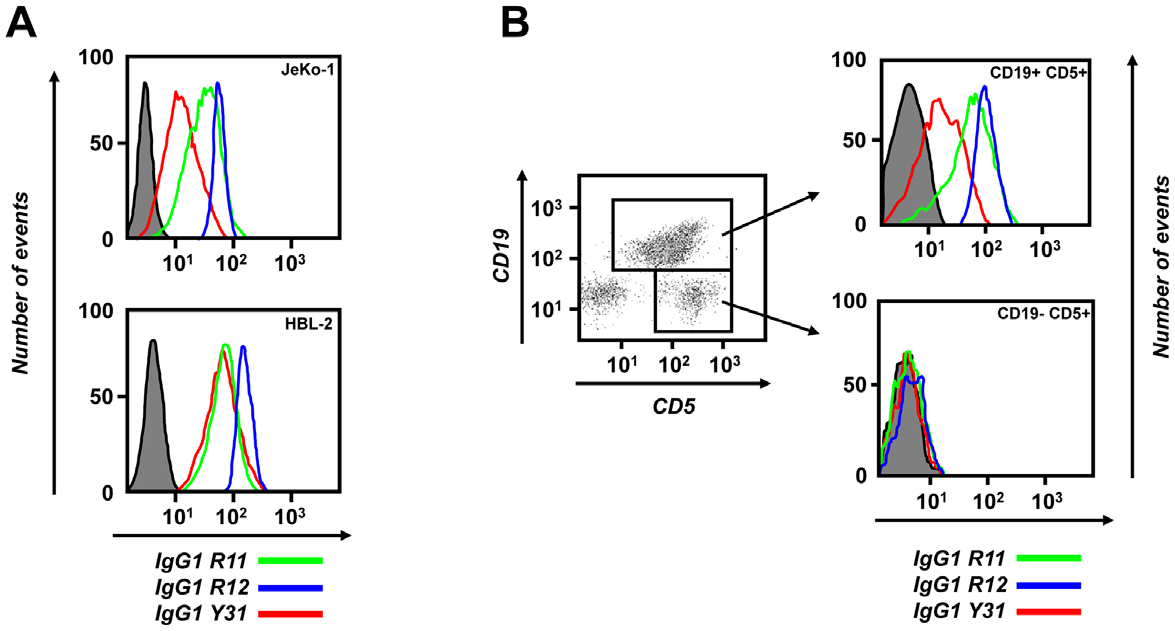
Cell surface binding. Flow cytometry was used to analyze the binding of IgG1 R11 (green line; 5 µg/mL), R12 (blue line; 1 µg/mL), and Y31 (red line; 5 µg/mL) to (**A**) the surface of JeKo-1 (top) and HBL-2 cells (bottom) and (**B**) PBMC from patient CLL-1. The gray shade indicates the background observed with TT11 (**A**; 5 µg/mL) or P14 (**B**; 5 µg/mL). Biotinylated IgG1 alone (**A**) or in combination with FITC-CD19/APC-CD5 (**B**) were detected with PE-streptavidin. The *y* axis depicts the number of events, the *x* axis the fluorescence intensity.

**Figure 7:**
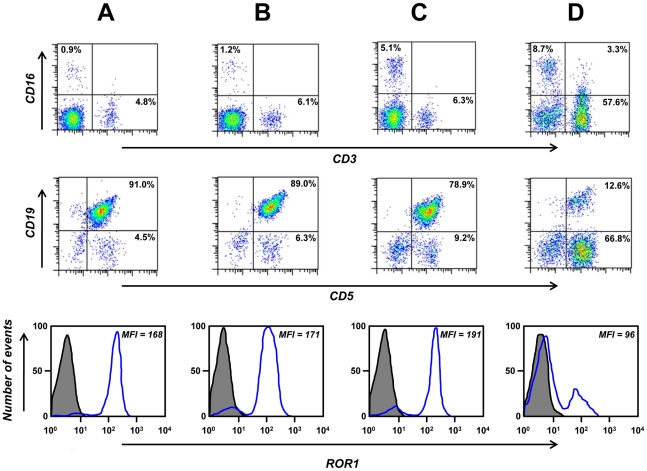
Specificity of cell surface binding. Flow cytometry was used to identify PBMC subpopulations from patients CLL-2 (**A**), CLL-3 (**B**), CLL-4 (**C**), and CLL-5 (**D**) as NK cells (CD16+ CD3−), T cells (CD16− CD3+ and CD19− CD5+), and CLL cells (CD19+ CD5+). The binding of biotinylated IgG1 R12 (blue; 1 µg/mL) to total PBMC is shown at the bottom. The gray shade indicates the background observed with PE-streptavidin alone. The *x* and *y* axes in the top and middle row and the *x* axis in the bottom row depict the fluorescence intensity, the *y* axis in the bottom row the number of events.

As mentioned before, Fab R11, R12, and Y31 recognized cell surface human ROR1 expressed by stably transfected HEK 293F cells (**Table 1**). Using flow cytometry, we validated the selective binding of IgG1 R11, R12, and Y31 to human MCL cell lines JeKo-1 and HBL-2 (**Fig. 6A**). Human anti-tetanus toxoid mAb TT11 in IgG1 format [31] was used as negative control. IgG1 R12 demonstrated strong and homogeneous (narrow peak) binding at concentrations as low as 0.01 µg/mL (67 pM) (data not shown), confirming its subnanomolar avidity found by surface plasmon resonance. By contrast, the binding of IgG1 R11 and, in particular, Y31 was somewhat weaker and more heterogeneous (broader peak). Although this pattern correlates with the different avidities found for the three mAbs (**Table 2**), we attribute it to the accessibility of the three different epitopes on cell surface ROR1. The presumed membrane distal epitope of R12 at the conjunction of Ig and Fz domains may facilitate better access for the bulky IgG1 format than the presumed membrane proximal epitope of R11 and Y31 in the Kr domain and at the conjunction of Fz and Kr domains, respectively. In fact, conversion of R11 to the less bulky scFv-Fc format (∼110 kDa; two polypeptide chains) demonstrated significantly stronger binding at lower concentrations compared to the IgG1 format (∼150 kDa; four polypeptide chains) (data not shown). An obstructed accessibility of their epitopes may dictate a predominantly monovalent rather than bivalent binding of IgG1 R11 and Y31 to cell surface ROR1, offsetting their subnanomolar avidity. We next analyzed the binding of IgG1 R11, R12, and Y31 to peripheral blood mononuclear cells (PBMC) prepared from CLL patients. Representative flow cytometry plots from one CLL patient are shown in **Fig. 6B** and corresponding mean fluorescence intensity (MFI) values over a concentration range of 0.01 to 10 µg/mL are shown in **Table 3**. Consistent with our previous study that was based on goat anti-human ROR1 pAbs [20], IgG1 R11, R12, and Y31 selectively bound to CLL cells (CD19+ CD5+), but not to normal T cells (CD19− CD5+) or other normal leukocytes. This finding underscored the specificity of IgG1 R11, R12, and Y31 for human ROR1. The pattern of binding to primary CLL cells was similar to that noted for the JeKo-1 and HBL-2 cell lines; strong and homogeneous binding of IgG1 R12, weaker and more heterogeneous binding of IgG1 R11 and Y31. Chimeric rabbit/human IgG1 P14 against NgR2 served as negative control and did not reveal cell surface binding to primary CLL cells (**Fig. 6B**). Flow cytometry plots that show the binding of IgG1 R12 to PBMC prepared from additional four CLL patients are shown in **Fig. 7**. Gating for normal NK cells, T cells, and B cells in these CLL patients further confirmed the specificity of IgG1 R12 for CLL cells.

**Table 3:** Flow cytometry binding of IgG1 R11, R12, Y31, and control antibodies to PBMC from patient CLL-1.

	0.01 µg/mL	0.1 µg/mL	1 µg/mL	5 µg/mL	10 µg/mL
**IgG1 R11**	ND^1^	6.6^2^	18.1	64.9	137.7
**IgG1 R12**	36.4	89.4	97.9	121.8	ND
**IgG1 Y31**	ND	5.4	8.3	21.6	58.9
**IgG1 TT11**	4.9	4.8	7.2	7.3	7.3

1 Not determined.

2 Mean Fluorescence Intensity (MFI).

### CDC assays with chimeric rabbit/human IgG1

**Figure 8:**
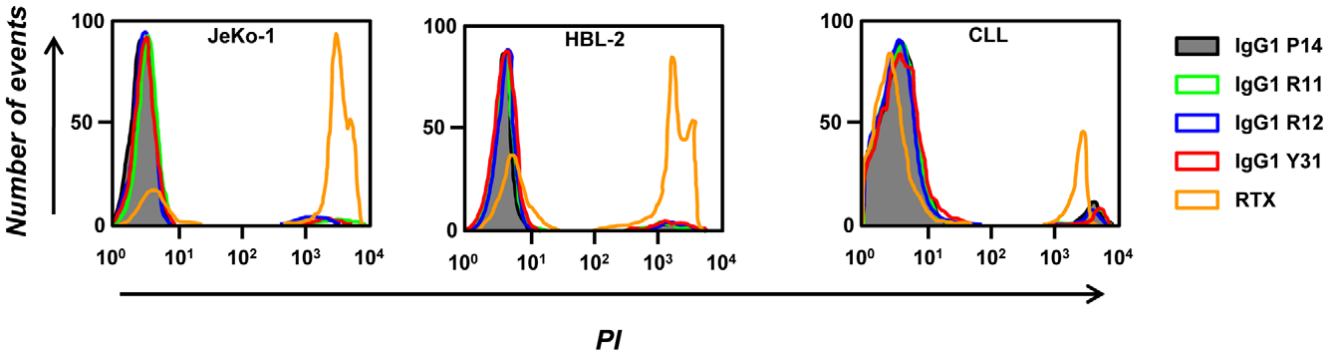
CDC. PI staining followed by flow cytometry was used to analyze the CDC potency of IgG1 R11, R12, and Y31 in comparison to IgG1 P14 (negative control), and rituximab (RTX; positive control; all at 10 µg/mL) toward JeKo-1 cells (left), HBL-2 cells (middle), and PBMC from patient CLL-6 (right) in the presence of rabbit complement. The percentage of dead cells (PI-positive) after RTX-mediated CDC was 70.7% (JeKo-1), 54.4% (HBL-2), and 12.7% (CLL).

Following confirmation of cell surface binding, we next investigated whether our chimeric rabbit/human IgG1 can kill cells expressing human ROR1 through complement fixation or CDC. As target cells we used human MCL cell lines JeKo-1 and HBL-2 in addition to PBMC prepared from CLL patients. Target cells were incubated with IgG1 R11, R12, Y31, P14 (negative control), and chimeric mouse/human anti-CD20 IgG1 mAb rituximab (positive control) followed by rabbit complement. Cell death was quantified by propidium iodide (PI) staining using flow cytometry. Whereas rituximab mediated potent CDC, none of the other antibodies revealed cytotoxicity above background (**Fig. 8**), and neither did a mixture of IgG1 R11 and R12 or rabbit anti-human ROR1 IgG pAbs purified from the serum of our immunized rabbits (data not shown). These findings suggested that ROR1 is not a suitable antigen for mediating CDC by mAbs or pAbs in IgG format regardless of epitope location.

### ADCC assays with chimeric rabbit/human IgG1

**Figure 9:**
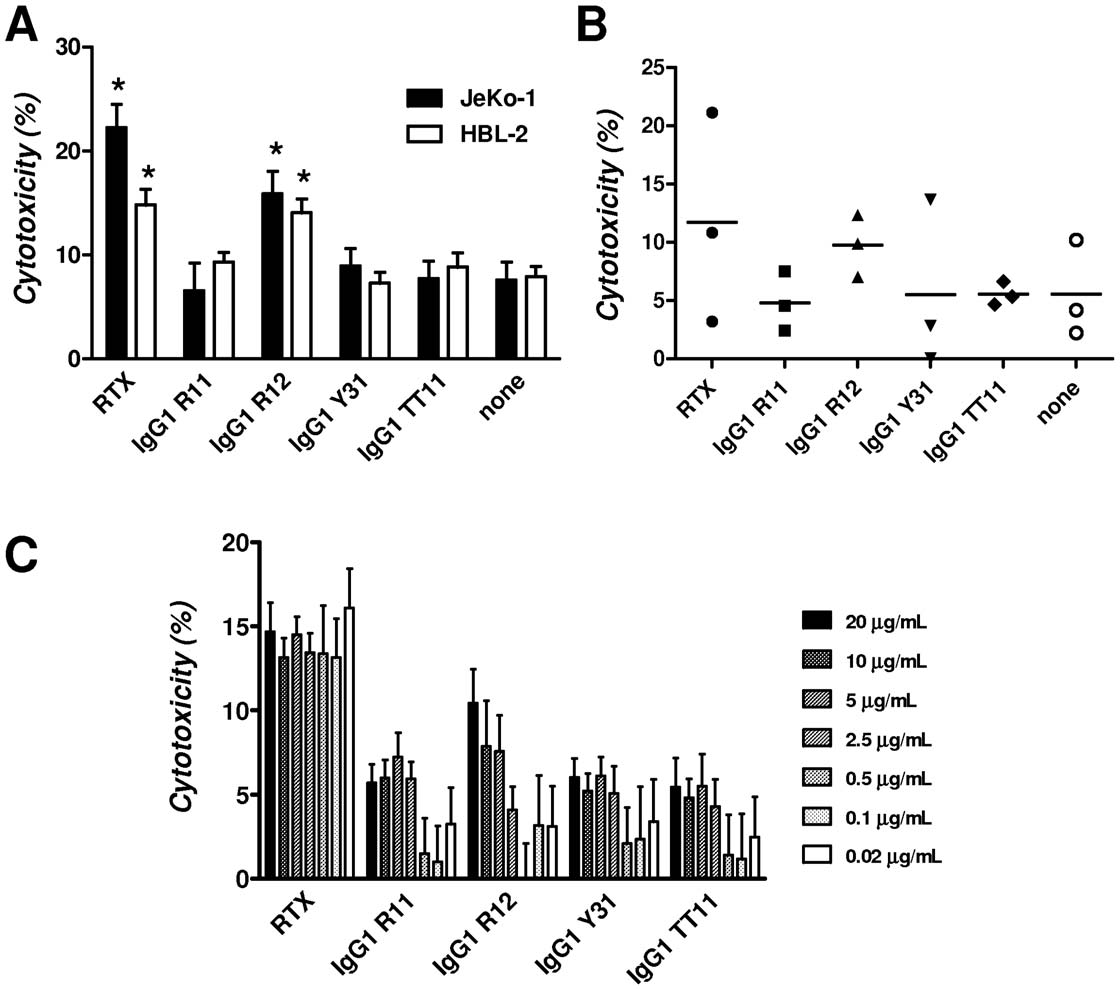
ADCC. A bioluminescent intracellular protease detection assay was used to quantify the ADCC potency of IgG1 R11, R12, and Y31 in comparison to IgG1 TT11 (negative control) and rituximab (RTX; positive control; all at 5 µg/mL) toward (**A**) MCL lines JeKo-1 and HBL-2 and (**B**) PBMC from patients CLL-2, CLL-3, and CLL-4. NK cells isolated from PBMC of a healthy volunteer were used as effector cells at an effector-to-target cell ratio of 25∶1. In (**A**), specific cytotoxicity is shown as columns with error bars to indicate mean and standard deviation values of side-by-side triplicates. Stars indicate significant differences (p<0.05) to both the negative control (IgG1 TT11) and the background (none). In (**B**), specific cytotoxicity toward PBMC from the three individual patients is shown with mean values indicated by horizontal bars. (**C**) Using the same assay and mAbs, ADCC potency toward HBL-2 cells was compared over a concentration range of 0.02 to 20 µg/mL. NK cells isolated from PBMC of a different healthy volunteer were used at an effector-to-target cell ratio of 20∶1. Columns indicate mean values and error bars indicate standard deviation values of side-by-side triplicates.

With CDC ruled out as a potential mechanism of killing human ROR1-expressing cells with our chimeric rabbit/human IgG1, we next investigated ADCC. As before, we used JeKo-1 and HBL-2 cells in addition to PBMC prepared from CLL patients as target cells. NK cells purified by negative magnetic selection from PBMC of a healthy volunteer were used as effector cells. An effector-to-target cell ratio of 25∶1 was chosen despite the fact that in CLL and leukemic MCL the number of circulating target cells is typically much higher than the number of circulating effector cells (**Fig. 7**). Using a bioluminescent intracellular protease detection assay, the killing of JeKo-1 and HBL-2 cells by IgG1 R11, R12, and Y31 in the presence of purified NK cells was quantified (**Fig. 9A**). IgG1 TT11 and rituximab were used as negative and positive control, respectively. At the chosen concentration of 5 µg/mL, only rituximab and IgG1 R12 mediated ADCC significantly above background toward both MCL cell lines. The same trend, although not significant, was found when PBMC from three CLL patients (**Fig. 7**) with 80% or more CD19+ CD5+ ROR1+ cells were used as target cells (**Fig. 9B**). Mixtures of IgG1 R11 and R12 did not reveal higher ADCC potency when compared to IgG1 R12 alone (data not shown). To further study their ADCC potency, we compared the same panel of mAbs over a concentration range of 0.02 to 20 µg/mL using the same assay with an effector-to-target cell ratio of 20∶1 (**Fig. 9C**). HBL-2 cells were used as target cells and purified NK cells from a different healthy volunteer as effector cells. Consistent with the previous experiments, IgG1 R12 was the only anti-ROR1 mAb that mediated ADCC at high concentrations. Nonetheless, its ADCC potency was vastly outdone by rituximab which demonstrated stable cytotoxicity over the entire concentration range (**Fig. 9C**). Although various protein and carbohydrate engineering strategies for improving the ADCC potency of mAbs exist, our findings argue against singling out ADCC as an effective strategy for killing human ROR1-expressing cells.

### Internalization of chimeric rabbit/human IgG1

**Figure 10:**
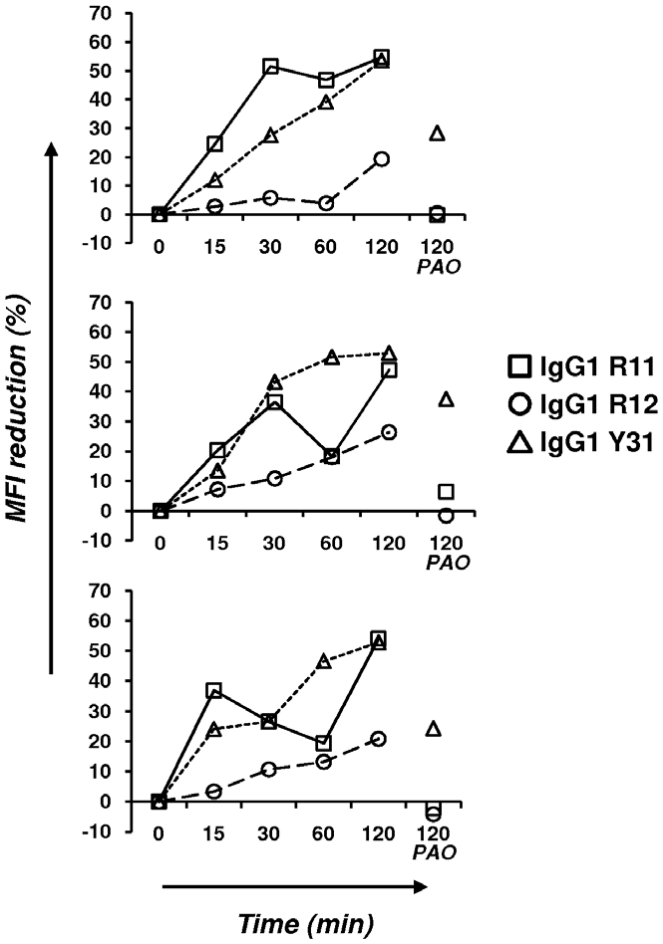
Internalization. Shown is the internalization of IgG1 R11, R12, and Y31 by PBMC from patients CLL-6 (top), CLL-7 (middle), and CLL-8 (bottom). Biotinylated IgG1 R11 (10 µg/mL), R12 (1 µg/mL), and Y31 (10 µg/mL) were incubated at 37°C and analyzed at the indicated time points with PE-streptavidin. The percentage of MFI reduction was calculated as described in Materials and Methods. The data set on the right shows MFI reduction in the presence of endocytosis inhibitor phenylarsine oxide (PAO).

We next investigated whether internalization or dissociation could account for the inability of IgG1 R11, R12, and Y31 to mediate effective CDC and ADCC. We had previously shown that human ROR1 can mediate internalization of polyclonal goat anti-human ROR1 IgG by a route that can be completely blocked by endocytosis inhibitor phenylarsine oxide [20]. PBMC from three CLL patients were incubated with biotinylated IgG1 R11, R12, and Y31 on ice, washed, and either left on ice or incubated at 37°C for 15 min, 30 min, 1 h, or 2 h. Subsequent staining with PE-streptavidin was used to detect biotinylated IgG1 that had remained at the cell surface. An MFI reduction was noted for all three IgG1 after 2 h (**Fig. 10**). MFI reduction can be explained by internalization or dissociation or a combination of both. In case of IgG1 R11 and R12, phenylarsine oxide completely blocked MFI reduction, revealing internalization as the dominating factor. By contrast, dissociation clearly contributed to the steady disappearance of IgG1 Y31 from the cell surface (**Fig. 10**). Internalization studies with JeKo-1 and HBL-2 cells gave very similar results (data not shown). Notably, IgG1 R12 internalized more slowly than IgG1 R11 with peaks at 20–25% after 2 h compared to 50–55%. It is possible that this difference in cell surface retention accounts for the different ADCC potencies of IgG1 R12 and R11.

### Apoptosis assays with chimeric rabbit/human IgG1

**Figure 11:**
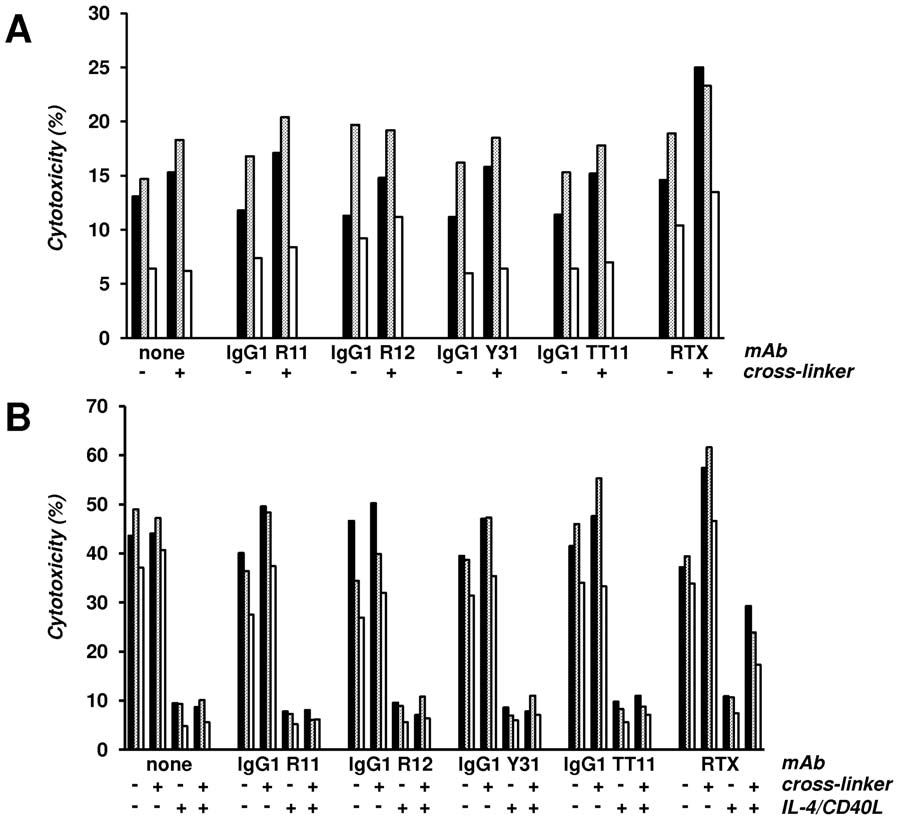
Apoptosis. PBMC from patients CLL-2 (black), CLL-3 (gray), and CLL-4 (white) cultured without (**A**) and with (**B**) FBS, were incubated with IgG1 R11, R12, Y31, TT11, and rituximab (RTX) for 72 h in the absence (−) and presence (+) of F(ab′)_2_ goat anti-human IgG (cross-linker). IL-4 and CD40L were added (+) in (**B**) to suppress apoptosis in the presence of FBS. Cytotoxicity was measured by separating live from apoptotic and dead cells by flow cytometry using Alexa Fluor 647 Annexin V and SYTOX Green nucleic acid stain.

The ability of IgG1 R11, R12, and Y31 to induce or inhibit apoptosis in primary CLL cells from patients was studied in the absence or presence of fetal bovine serum (FBS). FBS is known to enhance spontaneous apoptosis of primary CLL cells *ex vivo* [33]. Using FBS-free medium, we first investigated the induction of apoptosis in PBMC from three CLL patients (**Fig. 7**) with 80% or more CD19+ CD5+ ROR1+ cells following incubation for three days with IgG1 R11, R12, Y31, TT11, and rituximab alone or in the presence of a cross-linking pAb. As shown in **Fig. 11A**, the only increase in spontaneous apoptosis was noted for cross-linked rituximab. This was consistent and reproducible for all three PBMC samples tested. In the presence of FBS, apoptosis was approaching 50% after three days as expected (**Fig. 11B**). As observed previously [20], addition of IL-4 and CD40L strongly suppressed apoptosis. IgG1 R11, R12, Y31, and TT11 (negative control) neither increased nor decreased apoptosis alone or after cross-linking. They also did not influence the suppression of apoptosis by IL-4 and CD40L. By contrast, cross-linked rituximab was found to increase apoptosis and partially override its suppression (**Fig. 11B**). Collectively, this data demonstrates that our panel of chimeric rabbit/human IgG1 does neither induce nor inhibit apoptosis of primary CLL cells. We also investigated the induction of apoptosis in MCL cell line HBL-2 (data not shown). In contrast to primary CLL cells, rituximab alone was sufficient to induce apoptosis in HBL-2 cells. This activity was further increased after cross-linking. Nonetheless, IgG1 R11, R12, and Y31 did not induce apoptosis in HBL-2 cells with or without cross-linking.

## Discussion

Based on its favorable expression profile, receptor tyrosine kinase ROR1 is preclinically investigated as a new target for mAb therapy of CLL and MCL. Here we describe a panel of mAbs in chimeric rabbit/human Fab and IgG1 formats that were selected by phage display. This panel bound to a diverse set of epitopes covering all three domains in the extracellular region of human ROR1. Whereas the IgG1 format of this panel demonstrated comparable subnanomolar avidities for purified human ROR1, the distinct epitopes appeared to dictate substantially dissimilar cell surface binding and internalization characteristics. These distinctive properties were consistent for all cells we tested, including human MCL cell lines JeKo-1 and HBL-2 and primary CLL cells from different patients with variable cell surface densities of human ROR1.

Another distinctive property of the panel of selected mAbs to human ROR1 was cross-reactivity with mouse ROR1. Whereas R12 bound only human ROR1, R11 and Y31 recognized both human and mouse ROR1. We had previously shown that the selection of chimeric rabbit/human Fab libraries against conserved human antigens often yields mAbs that cross-react with their mouse and rat orthologs [25], [26], [27]. Human and mouse ROR1 share 97% amino acid sequence identity in the extracellular region. Notably, the Fz and Kr domains which harbor the epitopes of the cross-reacting mAbs R11 and Y31 are more conserved (99% amino acid sequence identity) than the Ig domain through the Fz domain conjunction (92% amino acid sequence identity) which harbors the epitope of the non-cross-reacting mAb R12. MAbs that recognize human and mouse ROR1 equally well are better suited for safety and activity assessments in xenogeneic and syngeneic mouse models and may allow a faster transition from preclinical to clinical studies as there is no need for developing surrogate mAbs. An analysis of publicly available genomic DNA sequences of the nonhuman primates *Pan troglodytes* and *Macaca mulatta* predicted that hROR1ECD shares 100% amino acid sequence identity with the corresponding chimpanzee and macaque protein (data not shown). Thus, mAbs and antibody derivatives that utilize the rabbit variable domains of R11, R12, and Y31 are suitable for preclinical toxicology studies in nonhuman primates.

The inability of our panel of chimeric rabbit/human IgG1 to mediate effective CDC and ADCC is likely a consequence of the relatively low cell surface density of ROR1. We previously determined that primary CLL cells express cell surface ROR1 in a range of 10^3^ to 10^4^ molecules per cell [20]. By comparison, our flow cytometry data suggest that MCL cell lines JeKo-1 and HBL-2 express roughly 1–2×10^4^ cell surface ROR1 molecules per cell. This is still one to two orders of magnitude below the typical cell surface density of antigens targeted by FDA-approved mAbs. Other investigational antigens in B-cell malignancies with cell surface densities comparable to ROR1, such as CD74 [34], were also found to be incapable of mediating ADCC and CDC. Even CD20, when expressed at relatively low cell surface densities of typically less than 5×10^4^ molecules per cell in CLL [35], may not mediate effective ADCC as suggested by the discovery that Fcγ receptor polymorphisms in CLL patients [36] do not influence the outcome of FC versus FCR therapy [37]. A likely mechanism of the clinical activity of rituximab in combination with chemotherapy is chemosensitization [38]. Although our anti-ROR1 mAbs did not induce apoptosis in primary CLL cells, they may still enhance the apoptotic activity of chemotherapy. Thus, it was important to demonstrate that our anti-ROR1 mAbs, unlike IL-4 and CD40L, do not provide detectable anti-apoptotic stimuli. MAbs to certain tyrosine kinase receptors, such as c-Met [39], [40], can exhibit undesired agonistic activity by mimicking the ligand and cross-linking the receptor.

Although our study suggests that naked mAbs to ROR1 may have limited therapeutic utility, it will be important to conduct *in vivo* studies as alternate mechanisms of activity, such as interference with yet unknown receptor/ligand interactions, cannot be assessed *in vitro* and *ex vivo*. In fact, the existence of endogenous anti-ROR1 antibodies that are triggered in CLL patients with clinical responses to an autologous cell vaccine [21] or lenalidomide [41] points to an alternate mechanism of activity. Relevant *in vivo* studies that may help to evaluate the therapeutic utility of naked anti-ROR1 mAbs will require mouse models that permit the transfer and proliferation of primary CLL or MCL cells. Nonetheless, we propose that ROR1 may be a preferred target for armed rather than naked mAbs. We recently demonstrated that ROR1-expressing primary cells and cell lines can be killed with T cells that express a ROR1-specific chimeric antigen receptor based on a mouse mAb to ROR1 [23]. It will be interesting to compare the potency of corresponding R11, R12, and Y31 chimeric antigen receptors as they provide a range of different affinities and epitopes. Our internalization data suggests that ROR1 may also be a suitable target for the delivery of cytotoxic payloads by antibody-drug conjugates and immunotoxins. It will be particularly interesting to compare the substantially dissimilar cell surface binding and internalization characteristics of R11 and R12 with respect to effective delivery of cytotoxic payloads.

In summary, we here describe a first generation of chimeric rabbit/human Fab and IgG1 that bind ROR1 with high affinity and specificity and provide both rationale and platform for a second generation of mAbs and antibody derivatives.

## Materials and Methods

### Primary cells and cell lines

Untreated CLL patients were enrolled in an institutional review board-approved observational study protocol at the Clinical Center, National Institutes of Health (NIH; Bethesda, MD) [42]. Written informed consent was obtained from all study participants. PBMC were prepared from blood by density gradient centrifugation using Lymphocyte Separation Medium (MP Biomedicals; Solon, OH) and used freshly or cryopreserved in liquid nitrogen until use. PBMC from healthy volunteers who gave written informed consent were prepared from fresh apheresis donations obtained from the Department of Transfusion Medicine, Clinical Center, NIH. Human NK cells (CD16+ CD56+) were negatively selected and purified from PBMC by magnetic activated cell sorting using the NK Cell Isolation Kit (Miltenyi Biotec, Bergisch Gladbach, Germany). Human MCL cell lines JeKo-1 and HBL-2 were obtained from American Type Culture Collection (Manassas, VA) and maintained in RPMI 1640 (Invitrogen; Carlsbad, CA) supplemented with 10% (v/v) heat-inactivated FBS (Thermo Scientific; Logan, UT), 100 U/mL penicillin, and 100 µg/mL streptomycin (Invitrogen). HEK 293F cells (Invitrogen) stably transfected with a mammalian expression vector (OriGene, Rockville, MD) that contained the full-length cDNA of human ROR1 (HEK 293F/hROR1) were described previously [31].

### Construction, expression, and purification of recombinant ROR1 proteins

***hROR1ECD*** **.** A cDNA fragment encoding a shortened extracellular region of human ROR1 (amino acids 51–391 + GK) with a V_H_ leader sequence was PCR-amplified with primers 5-K-sROR1ECD (gggtaccatggactggacctggagaatcctcttcttggtggcagcagccacaggagctcactccgaactcaacaaagattcttacctgac) and 3-B-sROR1ECD (gaggaggagggatccttatttaccgcacgctgggatgtcacacagatcagac) using ROR1 isoform 1 (GenBank accession no. NM_005012) TrueClone cDNA (OriGene) as template and was subsequently cloned via *Kpn*I/*BamH*I into mammalian expression vector pCEP4 (Invitrogen). Note that the C-terminus of hROR1ECD is identical to ROR1 isoform 2 (GenBank accession no. NM_001083592). ***Fc-hROR1*** **.** A cDNA fragment encoding the entire extracellular region of human ROR1 (amino acids 24–403) was custom synthesized by GenScript (Piscataway, NJ) and cloned into pCEP4 via *Hind*III/*BamH*I (pCEP4-hROR1). A genomic exon/intron sequence encoding human Fcγ1, equipped with a V_H_ leader sequence, a modified hinge region, and a modified C-terminus [43], was PCR-amplified with primers 5-K-FcX (gggtaccatggactggacctggaggatcctcttcttggtggcagcagccacaggagctcactccgagcccaaatcttctgacaaaactcacaca) and 3-H-FcX (cggagacaagcttaggctcttctgcgtgtagtggttgtgcag) and cloned into pCEP4-hROR1 via *Kpn* I/*Hind* III. ***Fc-mROR1*** **.** A cDNA fragment encoding a shortened extracellular region of mouse ROR1 (amino acids 51–391 + GK; GenBank accession no. NM_013845) was custom synthesized by DNA 2.0 (Menlo Park, CA) and cloned into pCEP4-Fc [43] via *Hind*III*/Xho*I. ***Fc-hROR1ig*** **.** A cDNA fragment encoding the Ig domain of human ROR1 (amino acids 51–147) was PCR-amplified with primers 5-H-Fc-ROR1ig (gagaagcttgtctccgggtgccgaactcaacaaagattcttacctgac) and 3-X-Fc-ROR1ig (gagctcgagttaaaacaagactccagtggaagaaacc) using ROR1 isoform 1 TrueClone cDNA as template and was subsequently cloned via *Hind* III*/Xho* I into pCEP4-Fc. ***Fc-hROR1fz*** **.** Analogous to Fc-hROR1ig, a cDNA fragment encoding the Fz domain of human ROR1 (amino acids 165–299) was cloned using primers 5-H-Fc-ROR1fz (gagaagcttgtctccgggtgccgaagaagatggattctgtcagcca) and 3-X-Fc-ROR1fz (gagctcgagttaaatccggatacagttcgcagcttc). ***Fc-hROR1kr*** **.** Analogous to Fc-hROR1ig, a cDNA fragment encoding the Kr domain of human ROR1 (amino acids 312–391 + GK) was cloned using primers 5-H-Fc-ROR1ki (gagaagcttgtctccgggtgccaagtgttataacagcacaggtgtgga) and 3-X-Fc-ROR1ki (gagctcgagttatttaccgcacgctgggatgtc). ***Fc-hROR1ig+fz***. Analogous to Fc-hROR1ig, a cDNA fragment encoding both Ig and Fz domain of human ROR1 (amino acids 51–299) was cloned using primers 5-H-Fc-ROR1ig and 3-X-Fc-ROR1fz. ***Fc-hROR1fz+kr***. Analogous to Fc-hROR1ig, a cDNA fragment encoding both Fz and Kr domain of human ROR1 (amino acids 165–391 + GK) was cloned using primers 5-H-Fc-ROR1fz and 3-X-Fc-ROR1kr. All eight constructs were transiently transfected into HEK 293F cells (Invitrogen) using 293fectin (Invitrogen) and conditions detailed in the manufacturer's protocol. Transfected cells were cultured in FreeStyle protein-free medium (Invitrogen) and the seven Fc fusion proteins of ROR1 were purified from supernatants by Protein A affinity chromatography as described [27]. In case of hROR1ECD, the supernatant was filtered through a 0.45-µm membrane and tenfold concentrated using an ultrafiltration device with a 10-kDa cutoff membrane (Millipore; Billerica, MA) and brought into buffer A (20 mM sodium phosphate, 25 mM NaCl, pH 7.4) using the same device. The concentrated sample was then purified by ion exchange chromatography using a 1-mL HiTrap Q FF column (GE Healthcare) and a 0–100% gradient to buffer B (20 mM sodium phosphate, 500 mM NaCl, pH 7.4). The eluted sample was brought into PBS using a 10-kDa cutoff centrifugal filter device (Millipore) and further purified by gel filtration chromatography using a Superdex 200 10/300 GL column (GE Healthcare). All chromatography procedures were carried out with an ÄKTA FPLC instrument (GE Healthcare). The quality and quantity of purified recombinant ROR1 proteins was analyzed by SDS-PAGE and absorbance at 280 nm, respectively.

### Generation and selection of chimeric rabbit/human Fab libraries

All rabbit handling was carried out by veterinary personnel at Spring Valley Laboratories, Inc. (Woodbine, MD). A total of six b9 allotype rabbits were used. Four rabbits were immunized and boosted three times with 100 µg Fc-hROR1, using Freund's complete and incomplete adjuvant (Sigma-Aldrich; St. Louis, MO) for two rabbits and TiterMax adjuvant (Sigma-Aldrich) for the other two rabbits. Library R was based on these four rabbits. Library Y was based on two additional rabbits that were immunized with 100 µg Fc-hROR1 and boosted three times with 100 µg hRORECD using Ribi (Sigma-Aldrich) adjuvant. Spleen and bone marrow from both femurs of each rabbit were collected five days after the final boost and processed for total RNA preparation and RT-PCR amplification of rabbit V_κ_, V_λ_, and V_H_ encoding sequences using established primer combinations and protocols [28]. Rabbit V_L_/human C_κ_/rabbit V_H_ segments were assembled in one fusion step based on 3-fragment overlap extension PCR, digested with *Sfi*I, and cloned into pC3C as described [28]. Transformation of E. coli strain XL1-Blue (Stratagene; La Jolla, CA) by electroporation yielded approximately 2.5×10^8^ and 1.4×10^8^ independent transformants for library R and Y, respectively. Using VCSM13 helper phage (Stratagene), the phagemid libraries were converted to phage libraries and selected by panning against immobilized protein as described [28]. Library R and Y were selected in parallel by four rounds of panning against hROR1ECD. Library Y was also selected by three rounds of panning on hROR1ECD followed by a final panning round on Fc-hROR1. During the panning against immobilized Fc-hROR1, unspecific polyclonal human IgG (Thermo Scientific; Rockford, IL) were added as decoy at a final concentration of 1 µg/µL. Supernatants of IPTG-induced selected clones were analyzed by ELISA using immobilized hROR1ECD and Fc-hROR1 and by flow cytometry using HEK 293F cells stably transfected with human ROR1 [31]. A C-terminal hemagglutinin (HA) decapeptide tag encoded on pC3C was used for detection as described below. Repeated clones were identified by DNA fingerprinting with *Alu*I, and the V_L_ and V_H_ sequences of unique clones were determined by DNA sequencing as described [28].

### Expression and purification of chimeric rabbit/human Fab

To remove HA tag and gene III fragment encoding sequences of pC3C [27] and add a C-terminal (His)_6_ tag, the expression cassettes encoding Fab R11, R12, and Y31 were transferred by *Sfi*I cloning into a Fab-(His)_6_ expression cassette in vector pET11a with an IPTG-inducible T7 promoter [44]. Following transformation into *E. coli* strain BL21-CodonPlus(DE3)-RIL (Stratagene) and expression through IPTG induction, Fab R11, R12, and Y31 were purified from bacterial supernatants by Immobilized Metal Ion Affinity Chromatography using a 1-mL HisTrap column (GE Healthcare) as described [45] followed by gel filtration chromatography using a Superdex 200 10/300 GL column with an ÄKTA FPLC instrument (GE Healthcare). The quality and quantity of purified Fab was analyzed by SDS-PAGE and absorbance at 280 nm, respectively.

### Expression, purification, and biotinylation of chimeric rabbit/human IgG

For the expression of R11, R12, and Y31 in IgG1 format, the previously described vector PIGG was used [25]. In this vector, γ1 heavy and κ light chain are expressed by an engineered bidirectional CMV promoter cassette. The V_H_ encoding sequences of Fab R11 and R12 were PCR amplified using primers R11-VH-5′(gaggaggagctcactcccagtcggtgaaggagtccga) and P14-VH-5′[27], respectively, in combination with R11-12-VH-3′(ccgatgggcccttggtggaggctgaggagatggtgaccagggtgcctggtccccagatg), and cloned via *Apa*I/*Sa*cI into PIGG. The light chain encoding sequences of Fab R11 and R12 were PCR amplified using primers P14-light-5′ [27] and R12-light-5′(gaggagaagcttgttgctctggatctctggtgcctacggggaactcgtgctgactcagtc), respectively, in combination with primer C-kappa-3′ [27], and cloned via *Hind*III/*Xba*I into PIGG with the corresponding heavy chain encoding sequence. Note that the resulting chimeric rabbit/human light chain of R12 is composed of a rabbit V_λ_ and a human C_κ_ domain. The V_H_ encoding sequence of Fab Y31 was PCR amplified using primers M5-VH-5′ and M5-VH-3′ [27], and cloned via *Apa*I/*Sac*I-ligation into PIGG. To remove an internal *Hind*III site by silent mutation, two fragments of the light chain encoding sequence of Fab Y31 were PCR amplified using primers P14-light-5′ in combination with Y31-light-3′(attggatgcataatagatcagtagcttgggaggctg) and Y31-light-5′(aaccagggcagcctcccaagctactgatct) in combination with C-kappa-3′, fused by overlap extension PCR using primers P14-light-5′ and C-kappa-3′, and cloned via *Hind*III/*Xba*I into PIGG with the corresponding heavy chain encoding sequence. The resulting PIGG-R11, PIGG-R12, and PIGG-Y31 plasmids were transiently transfected into HEK 293F cells (Invitrogen) using 293fectin (Invitrogen), and purified with a 1-mL recombinant Protein A HiTrap column (GE Healthcare, Piscataway, NJ) as described [27]. The quality and quantity of purified IgG1 was analyzed by SDS-PAGE and A_280_ absorbance, respectively. Chimeric rabbit/human IgG1 P14, which recognizes the LRRCT-unique fragment of human, rat, and mouse Nogo-66 receptor family member NgR2 [27], was expressed and purified as described previously [27] and used as negative control. Purified chimeric rabbit/human IgG1 was biotinylated using the BiotinTag Micro Biotinylation kit (Sigma-Aldrich). Briefly, 200 µg IgG1 in 50 µL of 0.1 M sodium phosphate buffer (pH 7.2) was incubated with 2 µL of 5 mg/mL biotinamidohexanoic acid 3-sulfo-N-hydroxysuccinide ester for 30 min at room temperature with gentle stirring. Biotinylated IgG1 was isolated using MicroSpin G-50 Columns provided by the kit.

### ELISA

For coating, each well of a 96-well Costar 3690 plate (Corning; Corning, NY) was incubated with 100 ng antigen in 25 µL TBS for 1 h at 37°C. After blocking with 150 µL 3% (w/v) BSA/TBS for 1 h at 37°C, 50 µL 500 ng/mL purified IgG1 (25 ng/well) in 1% (w/v) BSA/TBS was added, incubated for 2 h at 37°C, washed with H_2_O (10×200 µL/well), and incubated with 50 µL of a 1∶1,000 dilution of goat anti-human kappa pAbs conjugated to horseradish peroxidase (HRP) (Southern Biotech; Birmingham, AL) in 1% (w/v) BSA/TBS for 1 h at 37°C. Washing with H_2_O was repeated and colorimetric detection was performed using 2,2′-azino-bis(3-ethylbenzthiazoline)-6-sulfonic acid (Roche; Indianapolis, IN) as substrate according to the manufacturer's directions. In the same ELISA, 50 µL 500 ng/mL goat anti-human ROR2 pAbs (R&D Systems, Minneapolis, MN) followed by 50 µL of a 1∶1,000 dilution of donkey anti-goat IgG (H+L) pAbs conjugated to HRP (Jackson ImmunoResearch; West Grove, PA) were used as control. For the ELISA with bacterial supernatants after library selection, rat anti-HA mAb 3F10 conjugated to HRP (Roche) was used at a concentration of 50 ng/mL. The absorbance was measured at 405 nm using a VersaMax microplate reader (Molecular Devices; Sunnyvale, CA) and SoftMax Pro software (Molecular Devices).

### Surface plasmon resonance

Surface plasmon resonance for the measurement of the affinities of Fab R11, R12, and Y31 and the virtual affinities of IgG1 R11, R12, and Y31 to Fc-hROR1 and Fc-mROR1 as well as for epitope mapping studies were performed on a Biacore ×100 instrument using Biacore reagents and software (GE Healthcare). For affinity measurements, CM5 sensor chips were activated for immobilization with 1-ethyl-3-(3-dimethylaminopropyl)carbodiimide hydrochloride and N-hydroxysuccinimide. Fc-hROR1 and Fc-mROR1 fusion proteins in 10 mM sodium acetate (pH 5.0) were immobilized at a density of 669 resonance units (RU) for Fc-hROR1 and 429 RU for Fc-mROR1 in two flow cells on separate sensor chips. Subsequently, the sensor chips were deactivated with 1 M ethanolamine hydrochloride (pH 8.5). Each sensor chip included an empty flow cell for instantaneous background depletion. All binding assays used 1× HBS-EP+ running buffer (10 mM HEPES, 150 mM NaCl, 3 mM EDTA (pH 7.4), and 0.05% (v/v) Surfactant P20) and a flow rate of 30 µL/min. Fab and IgG1 R11, R12, and Y31 were injected at five or six different concentrations ranging from 1.5 to 100 nM in duplicates. The sensor chips were regenerated with glycine-HCl (pH 2.0) without any loss of binding capacity. Calculation of association (*k*_on_) and dissociation (*k*_off_) rate constants was based on a 1∶1 Langmuir binding model. The equilibrium dissociation constant (*K*_d_) was calculated from *k*_off_/*k*_on_. For epitope mapping studies, Fc-hROR1 was immobilized on a CM5 sensor chip at a density of 219 RU. IgG1 R11, R12, and Y31 were prepared as 300 nM solution in 1× HBS-EP+ running buffer. In the first cycle, IgG1 R11 was injected first, followed by a mixture of IgG1 R11 and R12, followed finally by a mixture of IgG1 R11, R12, and Y31. IgG1 R11 or IgG1 R11 in combination with IgG1 R12 were included in these mixtures to prevent signal loss due to dissociation. In the second cycle the injection order was R11, R11+Y31, and R11+Y31+R12. Analogously, R12 was injected first in the third and fourth cycle, and Y31 was injected first in the fifth and sixth cycle. RU increases that exceeded the values found for IgG1 R11, R12, and Y31 alone indicated independent epitopes that allow simultaneous binding.

### Flow cytometry

Cells were stained using standard flow cytometry methodology. Briefly, for anti-ROR1 Fab, cells were stained with unpurified or purified (5 µg/mL) Fab on ice for 1 h. After washing twice with ice-cold flow cytometry buffer (PBS containing 1% (v/v) FBS), cells were incubated with 5 µg/mL of biotinylated rat anti-HA mAb 3F10 (Roche) in flow cytometry buffer on ice for 1 h, washed as before, and stained with 2 µg/mL PE-streptavidin (BD Biosciences) on ice for 30 min. For anti-ROR1 IgG1, cells were first blocked with 100 µg/mL unspecific polyclonal human IgG at room temperature for 20 min, then incubated on ice for 1 h with different concentrations (0.01–10 µg/mL) biotinylated anti-ROR1 IgG1 alone (for HEK 293F/hROR1, JeKo-1, and HBL-2 cells) or in combination with FITC-CD19/APC-CD5 and FITC-CD3/APC-CD16 (BD Biosciences; Franklin Lakes, NJ) (for PBMC from CLL patients). After washing twice with ice-cold flow cytometry buffer, cells were stained with 2 µg/mL PE-streptavidin on ice for 30 min. PI was added to a final concentration of 5 µg/mL to exclude dead cells from analysis. Cells were analyzed using a FACSCalibur instrument (BD Biosciences) and FlowJo analytical software (Tree Star, Ashland, OR).

### CDC

JeKo-1 and HBL-2 cells or cryopreserved PBMC from CLL patients were harvested, washed, and resuspended in RPMI 1640 containing 10% (v/v) FBS, 100 U/mL penicillin, and 100 µg/mL streptomycin, and distributed into 96-well U-bottom plates (Corning) at a density of 1×10^5^ cells/well. After incubation for 1 h on ice with 10 µg/mL IgG1 R11, R12, Y31, negative control P14, or positive control rituximab (Genentech; South San Francisco, CA), the cells were harvested, washed once with PBS to remove unbound antibodies, and incubated with 20% complement from 3–4-week-old rabbits (Pel-Freez; Rogers, AR) for 2 h at 37°C in 5% CO_2_. After adding PI to a final concentration of 5 µg/mL, dead cells were detected by PI accumulation using a FACSCalibur instrument and FlowJo analytical software.

### ADCC

ADCC was assayed in a bioluminescent protease release assay (Glo Cytotoxicity Assay; Promega, Madison, WI) using the manufacturer's protocol with minor modifications. NK cells isolated from PBMC of healthy volunteers by magnetic activated cell sorting (as described before) were used as effector cells. JeKo-1 and HBL-2 cells or cryopreserved PBMC from CLL patients were used as target cells and distributed into 96-well U-bottom plates at a density of 1×10^4^ cells/well. The target cells were preincubated for 1 h at 37°C with serially diluted (from 20 to 0.02 µg/mL) IgG1 R11, R12, Y31, TT11 (negative control), or rituximab (positive control). Without washing, effector cells were added (100 µL/well) at an effector-to-target cell ratio of 20∶1 or 25∶1 and incubated for 24 h at 37°C in 5% CO_2_. After centrifugation, 50 µL/well of supernatant was transferred to a 96-well white clear-bottom Costar 3610 plate (Corning) followed by addition of 25 µL/well CytoTox-Glo cytotoxicity assay reagent (Promega, Madison, WI). After 15 min at room temperature, luminescence was measured with a Spectra Max M5 microplate reader (Molecular Devices, Sunnyvale, CA). The percentage of specific cytotoxicity was calculated according to the formula: Percent specific cytotoxicity = 100×(EX−*E*_spon_−*T*_spon_)/(*T*_max_−*T*_spon_), where EX represents the release from experimental wells, *E*_spon_ is the spontaneous release of effector cells alone, *T*_spon_ is the spontaneous release of target cells alone, and *T*_max_ is the maximum release from target cells lysed in 30 µg/mL digitonin. Data was computed as mean ± standard deviation of triplicates. Probabilities (p) were determined with Prism 5 software (GraphPad Software, La Jolla, CA) using the one-way ANOVA-Bonferroni's multiple comparison test; p<0.05 was considered significant.

### Internalization

Using a 96-well U-bottom plate, 3×10^6^ cryopreserved PBMC from CLL patients were first blocked with 100 µg/mL unspecific polyclonal human IgG at room temperature for 20 min, then stained with 10 µg/mL biotinylated IgG1 R11 and Y31, or 1 µg/mL biotinylated IgG1 R12 on ice for 1 h. Different concentrations were chosen to achieve similar levels of cell surface binding. After washing three times with flow cytometry buffer to remove unbound antibody, the cells were either left on ice or incubated at 37°C for 15 min, 30 min, 1 h, or 2 h to facilitate internalization. For the 2-h time point, duplicate samples were incubated in the absence or presence of 10 µM phenylarsine oxide (Sigma-Aldrich). Subsequently, the cells were washed once with flow cytometry buffer and incubated with PE-streptavidin on ice for 30 min. After three final washes with flow cytometry buffer, the MFI of the cells was measured using a FACSCalibur instrument and FlowJo analytical software. The percentage of MFI reduction was calculated for each mAb relative to the unspecific polyclonal human IgG control (MFI_background_) and mAb maintained on ice (MFI_max_) by using the formula [(MFI_max_−MFI_background_)−(MFI_experimental_−MFI_background_)]/(MFI_max_−MFI_background_)×100.

### Apoptosis

PBMC from CLL patients were distributed into 48-well flat-bottom plates at a density of 5×10^5^ cells/well in either (i) serum-free AIM-V medium (Invitrogen) supplemented with 50 µM β-mercaptoethanol (Sigma-Aldrich) or (ii) RPMI 1640 supplemented with 10% (v/v) heat-inactivated FBS, 100 U/mL penicillin, and 100 µg/mL streptomycin in the presence or absence of 100 ng/mL recombinant human IL-4 (R&D Systems) and 1 µg/mL soluble recombinant human CD40L trimer (Amgen, Thousand Oaks, CA). Cells were incubated with 5 µg/mL IgG1 R11, R12, Y31, TT11, or rituximab at 37°C in 5% CO2. For cross-linking, 20 µg/mL F(ab′)_2_ goat anti-human IgG (Fc-specific, Jackson ImmunoResearch Laboratories) was added to the cell suspension simultaneously with primary antibodies. Apoptosis and cell death was measured by flow cytometry following staining with Alexa Fluor 647 Annexin V (Invitrogen) and SYTOX Green nucleic acid stain (Invitrogen). Briefly, cells were gently harvested after 72-h incubation with indicated treatments, washed once with cold apoptosis binding buffer (140 mM NaCl, 2.5 mM CaCl_2_, 10 mM HEPES, pH 7.4), and resuspended in 200 µL apoptosis binding buffer. After adding 1 µL Alexa Fluor 647 Annexin V and 1 µL SYTOX Green to a final concentration of 50 nM, the cells were incubated for 15 min in the dark at room temperature, resuspended in 400 µL apoptosis binding buffer, and analyzed using a FACSCalibur instrument and FlowJo analytical software.
